# The Complement System in Flavivirus Infections

**DOI:** 10.3389/fmicb.2017.00213

**Published:** 2017-02-14

**Authors:** Jonas N. Conde, Emiliana M. Silva, Angela S. Barbosa, Ronaldo Mohana-Borges

**Affiliations:** ^1^Laboratório de Genômica Estrutural, Instituto de Biofísica Carlos Chagas Filho, Universidade Federal do Rio de JaneiroRio de Janeiro, Brazil; ^2^Laboratório de Bacteriologia, Instituto ButantanSão Paulo, Brazil

**Keywords:** complement system, flavivirus, dengue, NS1 protein, immune evasion

## Abstract

The incidence of flavivirus infections has increased dramatically in recent decades in tropical and sub-tropical climates worldwide, affecting hundreds of millions of people each year. The *Flaviviridae* family includes dengue, West Nile, Zika, Japanese encephalitis, and yellow fever viruses that are typically transmitted by mosquitoes or ticks, and cause a wide range of symptoms, such as fever, shock, meningitis, paralysis, birth defects, and death. The flavivirus genome is composed of a single positive-sense RNA molecule encoding a single viral polyprotein. This polyprotein is further processed by viral and host proteases into three structural proteins (C, prM/M, E) and seven non-structural proteins (NS1, NS2A, NS2B, NS3, NS4A, NS4B, NS5) that are involved in viral replication and pathogenicity. The complement system has been described to play an important role in flavivirus infection either by protecting the host and/or by influencing disease pathogenesis. In this mini-review, we will explore the role of complement system inhibition and/or activation against infection by the *Flavivirus* genus, with an emphasis on dengue and West Nile viruses.

## Introduction

The *Flaviviridae* family consists of many viral pathogens that cause severe disease and mortality in humans and animals. The family consists of four genera: *Flavivirus*, *Pestivirus*, *Pegvirus*, and *Hepacivirus*. In this mini-review, we will focus on the *Flavivirus* genus, which is the largest of the four genera, comprising more than 70 viruses, including dengue (DENV), West Nile (WNV), Zika (ZIKV), Japanese encephalitis (JEV), yellow fever (YFV), St. Louis encephalitis (SLEV), and tick-borne encephalitis (TBE) viruses (Lindenbach and Rice, 2003). The flaviviruses are primarily transmitted by arthropods such as mosquitoes and ticks. The most common mode of biological transmission is infection during a viremic blood meal and injection of infectious saliva during blood feeding (Kuno and Chang, 2005).

The rate of flavivirus infections worldwide has increased dramatically in the last few years, with the resurgence of all DENV serotypes (DENV1-4), WNV, YFV, and the emergence of ZIKV, affecting hundreds of millions of people each year (Gubler, 1998, 2002, 2007; Bhatt et al., 2013; Chan, 2016; Musso and Gubler, 2016). The large geographical expansion of flaviviruses has been related to vector adaptation of *Aedes aegypti* and *A. albopictus* to domesticated environments (Kuno and Chang, 2005). Other factors such as climate and genetic changes, global growth of human populations with extensive urbanization, lack of effective mosquito control, and increased air travel have also been associated with the emergence of flaviviruses (Gubler, 2002; Bennett et al., 2003; Steel et al., 2010; Weaver and Reisen, 2010).

Although most people infected with flaviviruses develop asymptomatic infection, the clinical spectrum is broad. After incubation for 3–6 days, there is an abrupt onset of many symptoms including high fever, chills, headache, back and muscle aches, dizziness, anorexia, nausea, and vomiting (Gould and Solomon, 2008; Murphy and Whitehead, 2011). Patients infected with one of the four DENV serotypes can develop severe manifestations that are characteristic of dengue hemorrhagic fever (DHF) and dengue shock syndrome (DSS), such as vascular leakage, that can lead to hypovolemic shock, coagulopathy that can be accompanied by bleeding, organ impairment and death (Simmons et al., 2012). Severe disease is associated frequently with secondary infection with a different serotype and primary infections of infants born to DENV-immune mothers. At concentrations that do not reach the stoichiometric threshold necessary for neutralization, anti-flavivirus antibodies enhance infection in cells expressing Fc-γR, a phenomenon known as antibody-dependent enhancement (ADE; Halstead et al., 2005; Pierson et al., 2007). Together with two additional non-exclusive hypotheses of viral virulence and serotype-cross-reactive memory T cells, these three processes could lead to excessive complement activation and cytokine release, resulting in DHF/DSS (Balsitis et al., 2010; Zellweger et al., 2010; Rothman, 2011).

In addition, humans infected with WNV develop meningitis or encephalitis syndrome that could be fatal in immunocompromised patients (Granwehr et al., 2004), while ZIKV infections may cause neurological complications such as microcephaly in neonates and Guillain–Barré syndrome in adults (Musso and Gubler, 2016). Because these severe manifestations occur relatively late during the course of disease when the virus may no longer be detectable in plasma, mechanisms mediated by immune responses may play a significant role in pathogenesis (Simmons et al., 2012).

## Flavivirus Structure

Flavivirus virions are ∼500 Å in diameter and are composed of a single positive-strand RNA genome, that is packaged by the viral capsid protein (C) in a host-derived lipid bilayer and surrounded by 180 copies of two structural proteins, envelope (E) and membrane (M, which is expressed as the precursor prM; Lindenbach and Rice, 2003; Mukhopadhyay et al., 2005). The genome is translated into a single polyprotein, and subsequently cleaved by viral and host proteases into three structural proteins (C, prM/M, and E) and seven non-structural (NS) proteins (NS1, NS2A, NS2B, NS3, NS4A, NS4B, and NS5; Lindenbach and Rice, 2003). The E protein functions in receptor binding, viral entry, and membrane fusion and elicits most neutralizing antibodies, whereas prM assists in folding, assembly, and function of the E protein (Lindenbach and Rice, 2003). The NS proteins regulate RNA transcription and replication as well as evasion and attenuation of the host immune response (Lindenbach and Rice, 2003; Dalrymple et al., 2015). Viral particles assemble at the endoplasmic reticulum (ER) and are released by exocytosis following transport through the *trans*-Golgi network (Mukhopadhyay et al., 2005).

## Complement System and Flaviviruses

### Pathogenic Effects of Complement Activation during Flavivirus Infections

The complement system is composed of more than 50 plasma proteins and receptors. Several complement proteins found in the blood normally circulate as inactive precursors and, once activated, they initiate a proteolytic cascade, resulting in release of chemokines, opsonization, and deposition of the cell-killing membrane attack complex (MAC; Merle et al., 2015a). Activation of the complement system occurs via three convergent pathways, the classical, the lectin, and the alternative pathways. The classical pathway is initiated by C1q binding to antigen-antibody complexes on the surface of pathogens or by direct binding of C1q to pathogen protein, or to the virion itself (Thielens et al., 2002). The lectin pathway is triggered by mannose-binding lectin (MBL) or ficolin recognition of carbohydrates on cell surfaces. The alternative pathway is constitutively activated at low concentrations through spontaneous hydrolysis of C3 and serves to amplify activation triggered by the classical and lectin pathways (Merle et al., 2015a). Binding of C3b to C3 convertases generates C5 convertases, which cleave C5 into C5a and C5b. C5b initiates the assembly of the MAC by interacting with C6, C7, C8, and multiple C9 molecules in the membrane of the pathogen (Merle et al., 2015a).

The complement system has an antagonistic role in flavivirus infections, either by limiting viral replication and protecting the host or by causing an exacerbated inflammatory response when excessively activated, increasing disease severity (Shresta, 2012). The majority of studies have focused on the role of complement in the context of DENV and WNV pathogenesis. Initial *in vitro* studies showed that macrophage complement receptors (CR3) mediate IgM-dependent increase of flavivirus replication in the presence of complement, and a blockade of CR3 abolishes complement-dependent enhancement of WNV infection (Cardosa et al., 1983, 1986). During WNV neuroinvasive disease, patients that survive often exhibit memory impairment, an effect that was related to complement-mediated elimination of presynaptic terminals (Vasek et al., 2016). C1q was found upregulated and localized to microglia, infected neurons, and presynaptic terminals during WNV infection, and mice deficient in CR3 receptor were protected from WNV-induced synaptic terminal loss (Vasek et al., 2016).

Moreover, clinical and *in vivo* studies showed that excessive consumption of C3, C4, factor B and C5 contributed to DHF/DSS and increased levels of the products of complement activation (C3a, C5a) that contribute to histamine release, enhanced vascular permeability and vasodilatation both in DENV and WNV infections. Indeed, the anaphylatoxins concentration in the blood of severe patients correlated with symptoms of vascular leakage (Bokisch et al., 1973; Nishioka, 1974; Mehlhop and Diamond, 2006; Nascimento et al., 2009; Pinto et al., 2014). Furthermore, anti-DENV antibodies activated complement on the surface of endothelial cells resulting in MAC formation (Avirutnan et al., 1998). Another study showed that NS1, C3a, C5a and soluble C5b-9 were present at higher levels in the plasma of DHF patients before plasma leakage (Avirutnan et al., 2006), supporting the hypothesis that exacerbated complement activation influences disease severity. Moreover, gene expression of the complement inhibitor CD59 was up-regulated more strongly in peripheral blood mononuclear cells (PBMCs) from non-severe dengue patients than in DHF patients (Ubol et al., 2008). These studies suggest that complement activation, and further cytokine induction may be important factors that contribute to vascular leakage, which is characteristic of DHF/DSS pathogenesis, even though the underlying mechanisms are not well understood.

### Protective Effects of Complement Activation during Flavivirus Infections

The complement system plays a key role in protection against viruses by diverse mechanisms: direct inactivation of virions by MBL; recruitment and activation of monocytes and granulocytes by the anaphylatoxins C3a and C5a; opsonization of viral particles mediated by the proteolytic fragments of C3 (C3b, iC3b, C3d, and C3dg), which facilitate clearance by cells that express complement receptors; antigen uptake and presentation facilitated by C3; and lysis of enveloped viral particles and infected cells by the MAC (Stoermer and Morrison, 2011; Merle et al., 2015b).

Several studies have shown a protective effect for complement on flavivirus infection. *In vitro* assays assessed ADE of DENV infection and showed that fresh serum could abolish ADE (Mehlhop et al., 2007; Yamanaka et al., 2008). Further analysis demonstrated that this inhibitory effect was C1q-dependent both *in vitro* and *in vivo* and IgG subclass-specific, suggesting that complement proteins could limit disease progression (Mehlhop et al., 2007). In addition, C1q enhanced the neutralizing activity of anti-WNV antibodies, and reduced the number of antibodies required to bind the WNV virion and neutralize infectivity (Mehlhop et al., 2009b). Another study demonstrated that C1q binds directly to both the DENV E protein and the viral particle, and when C1q was pre-incubated prior to DENV infection of THP-1 cells, decreased viral infectivity was observed (Douradinha et al., 2014). Proteomics data confirmed the binding of several complement proteins, including C1q, C1s, C1r, C5, C9, and mannan-binding lectin serine protease 1 (MASP1) on viral-enriched fractions from DENV-infected patients and on domain III of the DENV E protein (Fragnoud et al., 2015; Huerta et al., 2016).

Moreover, *in vitro* studies reported that serum complement neutralizes both WNV in solution and WNV-infected cells in an antibody-dependent manner (Mehlhop et al., 2005). Neutralization by serum complement also occurred through direct binding of MBL, which activated MASP2 and resulted in further opsonisation of the virion surface, preventing viral fusion with host membranes (Fuchs et al., 2010). Furthermore, MBL but not C1q or C5 restricted infection of all DENV serotypes by both complement-dependent and independent mechanisms. Nevertheless, the neutralization was more efficient with insect-derived virus than mammalian-derived virus. Further analysis showed that MBL concentration in human blood correlated directly with DENV neutralization, indicating that polymorphisms in the MBL gene may have an impact on DENV infection and severity (Avirutnan et al., 2011a). In addition, *in vivo* studies suggested that all activation pathways were required to restrict WNV infection in mice (Mehlhop et al., 2005; Mehlhop and Diamond, 2006; Fuchs et al., 2011). However, WNV neutralization occurred primarily through C5- and MAC-independent mechanisms (Mehlhop et al., 2009a). Therefore, opsonization may be the main mechanism of flavivirus neutralization, as the efficiency of MAC formation may be limited by the small surface area of exposed flavivirus membrane.

### Complement Activation and Evasion by NS1 Protein

Among the NS proteins, NS1 is the primary flavivirus protein that is antagonistically related to both complement activation and evasion. NS1 is a highly conserved glycoprotein with a molecular weight of 46–55 kDa, depending on its glycosylation status. After polyprotein processing, NS1 is translocated into the lumen of the ER, released from E protein by ER resident signal peptidase (Muller and Young, 2013; Zhang et al., 2016), cleaved at its C-terminus by an unidentified ER host protease (Falgout and Markoff, 1995), and glycosylated by the addition of high-mannose carbohydrates (Pryor and Wright, 1994). After a rapid dimerization, NS1 acquires a partially hydrophobic behavior and associates with cell membranes. The atomic resolution of dimeric DENV and WNV NS1 structures revealed a hydrophobic region comprised of approximately the first 20 residues of each monomer that form the beta-roll domain (Akey et al., 2014). Together with another hydrophobic region 150 residues downstream, these domains are postulated to insert into membranes (Watterson et al., 2016). An alternative mechanism through which DENV NS1 interacts with cell membranes was described after the identification of a glycosyl-phosphatidylinositol (GPI)-linked form (Jacobs et al., 2000).

Thereafter, NS1 traffics to multiple destinations, including sites of viral replication complex and associated vesicle packets (VPs; Mackenzie et al., 1996; Lindenbach and Rice, 1997; Khromykh et al., 1999), the cell membrane (mNS1; Winkler et al., 1989; Schlesinger et al., 1990), and the extracellular environment through secretion (sNS1; Crooks et al., 1990, 1994; Flamand et al., 1999). The secreted form of NS1 traffics through the Golgi secretory pathway in mammalian cells, and the carbohydrate moieties are processed to more complex sugars that are then secreted as a soluble hexamer associated with lipids, with a molecular weight of ∼300 kDa (Crooks et al., 1990, 1994; Flamand et al., 1999; Gutsche et al., 2011). Secreted NS1 was found circulating in the blood of flavivirus-infected patients at high levels (Alcon et al., 2002) and servers as a primary biomarker for diagnosis.

The NS1 protein has been studied by several groups, and a wide variety of functions has been assigned to it. In the 1970s, a DENV soluble complement fixing antigen (SCF) that was later identified and confirmed to be NS1 was detected in infected mice and cell culture (Brandt et al., 1970; McCloud et al., 1970; Russell et al., 1970). Both mNS1 and sNS1 activated complement in the presence of anti-serum, and sNS1 triggered complement activation even in the absence of specific antibodies (Avirutnan et al., 2006). High levels of NS1, C5a, and SC5b-9 were present in pleural fluids from patients with DSS, and their presence correlated with disease severity (Avirutnan et al., 2006).

In addition to complement activation and auto-immune responses, co-administration of recombinant NS1 with DENV in an animal model of the disease resulted in morbidity, cytokine induction and endothelial barrier disruption (Beatty et al., 2015). Another recent study demonstrated that DENV NS1 binds TLR4 on the surface of CD14+ monocytes and induces cellular activation, cytokine production and vascular permeability, a similar response triggered by the bacterial endotoxin LPS (Modhiran et al., 2015). Therefore, the effects of NS1, combined with exacerbated immune responses, could contribute to the disruption of endothelial cell layer integrity, resulting in hemorrhage and vascular leakage, which are characteristic of dengue and YFV (Monath and Barrett, 2003; Basu and Chaturvedi, 2008). Such a combination may also contribute to deregulation of the blood brain barrier, which is a crucial component of the development of encephalitis caused by WNV and JEV (Neal, 2014).

**FIGURE 1:**
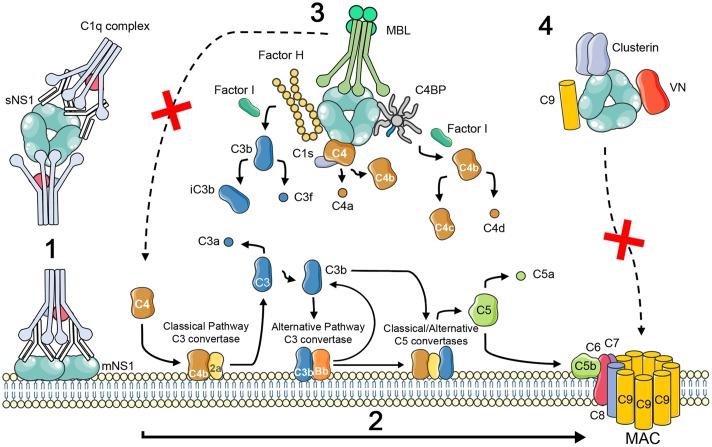
**Schematic illustration of complement activation and evasion by NS1 protein of flavivirus.** NS1 is expressed on the surface (mNS1) and is also secreted by flavivirus-infected cells (sNS1). Circulating sNS1 can subsequently bind to the surface of both infected and uninfected cells. In dengue virus secondary infections, IgM and IgG antibodies to NS1 during the acute phase of the disease can lead to the formation of immune complexes (1) that trigger inflammatory responses, including the activation of the complement cascade to generate the anaphylatoxins C3a and C5a, opsonins, and the membrane attack complex (MAC; reviewed by Muller and Young, 2013) (2). NS1 has also been shown to contribute to viral immune evasion through interaction with complement proteins and its regulators. Binding to the alternative complement pathway regulatory protein FH leads to accelerated breakdown of the alternative pathway convertase with consequent reduced C3b deposition and MAC formation (Chung et al., 2006). NS1 also binds to proC1s/C1s and C4 which results in increased cleavage of C4 to C4a and C4b, what may limit the amount of C4 available (Avirutnan et al., 2010). In addition, C4BP bound to NS1 can act as a cofactor for Factor I-mediated cleavage of C4b to C4c and C4d. Deposition of the sNS1–C4BP complex on the cell surface could lead to inactivation of cell surface bound C4b thereby protecting infected cells from complement mediated lysis (Avirutnan et al., 2011b). NS1 from DENV-infected insect cells binds MBL and may protect DENV from MBL-mediated neutralization, suggesting a role for limiting complement activation at the site of the mosquito bite (Thiemmeca et al., 2016) (3). NS1 was suggested to inhibit MAC formation by interacting with vitronectin (VN), clusterin, and the terminal complement protein C9 (Kurosu et al., 2007; Conde et al., 2016) (4).

In response to antiviral mechanisms, viruses have evolved specific strategies to avoid complement activation and neutralization by producing or binding complement-regulatory or complement-blocking molecules (Stoermer and Morrison, 2011; Merle et al., 2015b). Flavivirus NS1 has been described as an immune evasion protein that can attenuate activation of the classical, lectin and alternative pathways by interacting with complement proteins and their regulators (**Figure 1**). It is worth mentioning that these interactions were not confirmed experimentally for all flavivirus and may be specific for certain viruses. WNV NS1 has been shown to bind Factor H (FH), and bound FH retains cofactor activity for Factor I-mediated cleavage of C3b to iC3b. In addition, cell membrane-associated NS1 also recruits FH and consequently leads to a decreased deposition of C3b and the MAC on cell surfaces (Chung et al., 2006). DENV, WNV, and YFV also bind C4 and C1s/proC1s in a complex to cleave C4 to C4b in solution, thus reducing the deposition of C4b on cell surfaces and the activity of the classical C3 convertase (C4b2a) (Avirutnan et al., 2010). C4b Binding Protein (C4BP), which regulates both the classical and the lectin pathways of complement, was also recruited by DENV, WNV, and YFV NS1. Once bound to NS1, C4BP, as a cofactor for FI, mediates inactivation of C4b in solution and on the plasma membrane of cells (Avirutnan et al., 2011b). FH, C1s, and C4BP have cysteine-rich domains called complement control protein (CCP) domains. Some proteins from different pathogens have been shown to interact with CCP domains via an anti-parallel β-sheet motif (Persson et al., 2007; Schneider et al., 2009; Bhattacharjee et al., 2013). Thus the β-roll and β-ladder domains of NS1, which have an anti-parallel β-sheet folding, are proposed to be candidates for binding to complement proteins (Akey et al., 2015). Also, NS1 from DENV-infected insect cells binds MBL to protect DENV from MBL-mediated neutralization, suggesting a role for limiting complement activation at the site of mosquito bite (Thiemmeca et al., 2016).

Through a yeast two-hybrid approach using a human liver cDNA library, C1q was identified as a DENV NS1-binding partner (Silva et al., 2013). However, it is unclear whether this interaction contributes to complement activation or complement evasion. DENV sNS1 has also been reported to bind complement proteins from the terminal pathway. Clusterin, which inhibits the formation of the MAC, is among the NS1 targets (Kurosu et al., 2007), although the functional outcome of this interaction has not been explored. Recently, we demonstrated that DENV NS1 interacts directly with vitronectin (VN), a regulatory protein that hinders the insertion of the MAC into membranes and binds the terminal complement proteins C5, C6, C7, and C9 (Conde et al., 2016). DENV NS1 alone or in association with VN, inhibited C9 polymerization, thus preventing lytic pore formation independently from its glycosylation pattern. Moreover, by binding to C9, mammalian secreted DENV, WNV, and ZIKV NS1 inhibited MAC formation on cell membranes. Taken together, these data imply a role of NS1 as a terminal pathway inhibitor of the complement system (Conde et al., 2016). It is more likely that NS1 acts as a complement evasion protein during the acute phase of the disease when anti-NS1 antibodies have not yet formed immune complexes, providing a less adverse extracellular environment for the released viruses. In the context of dengue secondary infections, ADE, immune complex deposition and complement activation may be critical for the development of severe cases.

## Concluding Remarks

The complement system certainly influences the outcome of flavivirus infections. However, we still have only a glimpse of the mechanisms that orchestrate the protective effects of complement activation. On the other hand, there are consequences for the host that arise from complement downregulation by those viruses. Research in this area will contribute to the current knowledge of flavivirus pathogenesis, providing insight into the regulation of immune responses and leading to improved anti-viral inhibitors and vaccine approaches.

## Author Contributions

JC conceived of the review outline. JC researched and drafted the manuscript. ES, AB, and RM-B provided significant input to several sections to improve clarity and accuracy.

## Conflict of Interest Statement

The authors declare that the research was conducted in the absence of any commercial or financial relationships that could be construed as a potential conflict of interest.
